# Evaluating the Role of *Candida albicans* as a Potential Oral Carcinogen

**DOI:** 10.1155/ipid/4057977

**Published:** 2025-11-20

**Authors:** Fatemeh Amirinia, Marjan Motamedi, Pegah Ardi, Ahmad Jabrodini

**Affiliations:** ^1^Department of Medical Parasitology and Mycology, School of Medicine, Shiraz University of Medical Sciences, Shiraz, Iran; ^2^Dental Research Center, Dentistry Research Institute, Tehran University of Medical Sciences, Tehran, Iran; ^3^Department of Medical Parasitology and Mycology, School of Public Health, Tehran University of Medical Sciences, Tehran, Iran; ^4^Cellular and Molecular Research Center, Gerash University of Medical Sciences, Gerash, Iran

**Keywords:** *Candida albicans*, carcinogenesis, neoplasms, oral cavity, oral neoplasm

## Abstract

Oral cavity cancers, especially oral squamous cell carcinoma (OSCC), are a major subset of head and neck malignancies. Increasing evidence indicates that oral microbiota, particularly *Candida albicans* (*C. albicans*), plays a significant role in OSCC development and progression. While *C. albicans* is normally a commensal organism in the human microbiome, it can become pathogenic under certain conditions. The carcinogenic potential of *C. albicans* contributes to oral cancer by acting both as a co-factor and a direct pathogen. This involves complex interactions with the host immune system and the expression of multiple virulence factors. The dynamic relationship between *C. albicans* and its host influences disease outcomes and cancer progression. This review focuses on key virulence mechanisms of *C. albicans*, including adhesion to host cells, secretion of hydrolytic enzymes, production of carcinogenic metabolites, induction of chronic inflammation, and release of candidalysin, a cytolytic peptide toxin. Understanding these factors is essential to clarify how *C. albicans* promotes OSCC initiation and progression. Improved knowledge of *C. albicans* virulence may lead to targeted therapies, preventive strategies, and novel biomarkers for early detection, ultimately enhancing treatment outcomes and prognosis for patients with oral cavity cancers.

## 1. Introduction

Cancer is the second leading chronic noncommunicable disease and the third leading cause of death globally, representing a major public health concern both worldwide and in Iran. Annually, over 50,000 new cancer cases are diagnosed in Iran, with a rising trend observed in recent years [1]. According to the 2016 report from Iran National Cancer Registry (INCR), 124,833 new cancer cases were registered in the country [1]. Squamous cell carcinoma (SCC) of the oral cavity, pharynx, and larynx—collectively referred to as head and neck cancers—accounts for approximately 3% of these new cases [2]. Globally, head and neck squamous cell carcinoma (HNSCC) affected an estimated 900,000 new cases and caused 500,000 deaths in 2020 [3]. It ranks as the sixth most common cancer and the seventh leading cause of cancer-related mortality worldwide. Studies indicate that SCC constitutes the predominant type of oral cancer, accounting for an average of 70.05% of cases [3–5]. Despite advances in treatment modalities—including surgery, chemotherapy, radiotherapy, immunotherapy, and targeted therapies—the overall prognosis and survival rates for patients with various forms of oral squamous cell carcinoma (OSCC) remain unsatisfactory. This underscores the urgent need for improved therapeutic strategies and early detection methods to enhance patient outcomes [6].

Numerous studies have highlighted the diverse impacts of lifestyle factors on the etiology and progression of OSCC [7–11]. Major risk factors for OSCC include alcohol consumption, tobacco use, infection with human papillomavirus (HPV) and Epstein-Barr virus (EBV), a diet low in vitamin A, poor oral hygiene, and chronic oral trauma [7, 11]. Furthermore, the significant roles of other predisposing factors—such as the microbiome, environmental exposures, and contact with chemicals, pesticides, and heavy metals—must be acknowledged, as they can induce genetic and epigenetic alterations that contribute to carcinogenesis [10, 11]. Microorganisms can play a crucial role in cancer development by inducing chronic inflammation, synthesizing carcinogenic substances, compromising the integrity of the epithelial barrier, and disrupting immune system function [10, 12]. The most famous of them is the well-known HPV that has been discussed a lot because of its involvement in the development of oral cancers [10].

Additionally, in this regard, the role of the body's natural microbiome should also be considered [13]. It affects its host in various ways and under different conditions, such as modulating immune responses, maintaining epithelial barrier integrity, producing toxic metabolites and proteolytic enzymes that influence cellular signaling, and competing with pathogenic microorganisms; it can impact epithelial cells and inflammatory processes [14]. Such damage and the ensuing chronic inflammation can induce genetic alterations and oncogenic mutations in host cells, contributing to cancer development. Furthermore, in hosts with different lifestyles—such as alcohol or tobacco consumption, which can alter the composition and metabolism of the oral microbiome—these factors may suppress the host's immune response, elevate the production of inflammatory cytokines, promote antiapoptotic activity, and facilitate the secretion of carcinogenic substances. These effects not only increase the risk of cancer development but also influence its progression [15, 16].

One of the most important fungi in the oral cavity is *Candida* spp., which not only causes secondary infections in cancer patients, but can also play an important role in causing cancer [17]. In addition, studies have pointed out the role of *Candida *spp. invasion as an important risk factor for the malignant transformation of oral cancer [8, 17–20].

Among *Candida* spp., *C. albicans* is considered one of the most common oral microbes in OSCC, which is associated with the initiation of oral neoplasia and the development of OSCC [18, 20–22]. Of course, differentiating *C. albicans* in a healthy state from a pathogenic state has always been challenging. Therefore, it is necessary to identify the relationship between cancerous lesions and the prevalence of *C. albicans*, and to identify the factors that increase carcinogenesis. We aim to review the key virulence factors of *C. albicans* involved in oral carcinogenesis and explore their potential for targeted therapies and early detection in OSCC.

### 1.1. Data Sources and Search Strategy

Databases such as PubMed, Google Scholar, Scopus, EBSCO, and Science Direct were searched using keywords such as (“Cancer” OR “Oral Cancer” OR “Mouth Neoplasms” OR “Oral Carcinogenesis” OR “Oral Tongue Squamous Cell Carcinoma” OR “OSCC” OR “Oral Squamous Cell Carcinoma”) AND (“*Candida albicans*” OR “*Candida*” OR “Oral Microbiota”). The keyword searches were run from 30 December 1980 to 1 September 2025. A search using the specified keywords produced 3850 results, encompassing conference presentations, books and journal publications including original article, letters to the editor, short communications, reviews, and case reports. A total of 105 pertinent full-text articles were found after reviewing titles and abstracts. Eventually, 53 articles were included in the review. This review article, by examining these 68 articles and analyzing them, provides evidence that suggests a link between the presence of *C. albicans* in the oral cavity and an increased risk of oral cancer.

### 1.2. Mechanisms Involved in *C. albicans* Carcinogenesis

The role of *C. albicans* in the process of carcinogenesis has not yet been fully clarified. In general, the interaction of multiple factors, such as oral symbiosis, host factors, and virulence factors related to *Candida*, are among the most important factors involved in carcinogenesis [21, 23]. We have tried to mention some effective factors in this regard.

## 2. Oral Symbiosis

**Figure 1:**
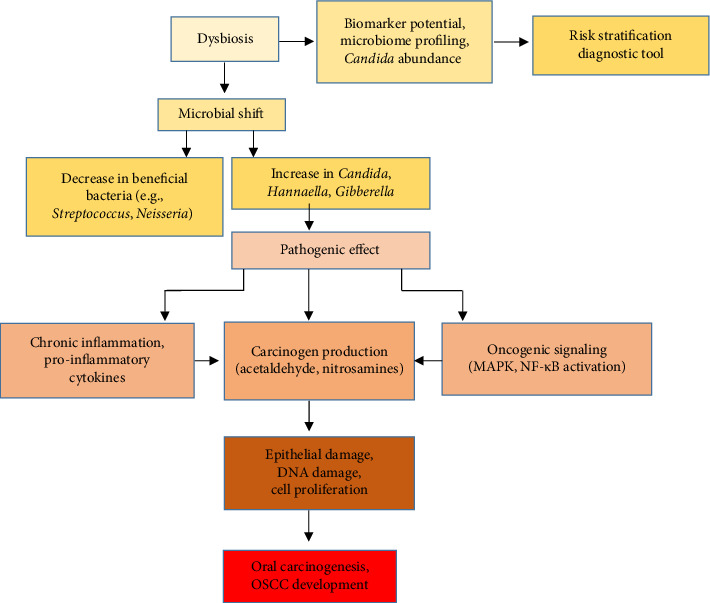
The role of dysbiosis and *Candida* in the development of squamous cell carcinoma.

The oral microbiome comprises a complex ecosystem of microorganisms that exist in a dynamic equilibrium, playing a crucial role in maintaining oral homeostasis and health [24, 25]. Under normal conditions, commensal microorganisms contribute to host defense by competing with pathogens, modulating immune responses, and preserving the integrity of the mucosal barrier. However, disruption of this delicate balance (known as dysbiosis) can lead to overgrowth of opportunistic pathogens such as *Candida* spp. [23]. Dysbiosis has been implicated in the pathogenesis of oral malignancies, with studies reporting significant alterations in the mycobiome of oral cancer patients. Notably, elevated abundances of *Candida*, *Hannaella*, and *Gibberella* have been observed in individuals with OSCC. These fungal shifts are often accompanied by reduced bacterial diversity and loss of beneficial taxa, further exacerbating inflammatory processes and epithelial damage. The persistence of *Candida* spp. in dysbiotic states promotes chronic inflammation, production of carcinogenic metabolites (e.g., acetaldehyde), and activation of pro-oncogenic signaling pathways [23, 26]. Consequently, the oral microbiome profile—particularly the relative abundance of *Candida* and allied genera—may serve as a promising diagnostic biomarker for early detection and risk stratification of oral premalignant and malignant lesions [26, 27] (Figure 1).

## 3. Host Factors

Key host-related factors influencing oral candidiasis include salivary gland dysfunction, use of certain medications, dentures, high-carbohydrate diets, and smoking. In healthy individuals, saliva contains antimicrobial substances such as lactoferrin, amylase, proline-rich glycoproteins (PRPs), lysozyme, and anti-*Candida* antibodies, which help prevent *Candida* spp. overgrowth and the development of oral candidiasis. Overgrowth of *Candida* spp. in these conditions contributes to oral cancer via inflammation, ulcerations, cellular alterations, and secretion of carcinogenic metabolites, as elaborated in this article [28]. Among genetic factors, gain-of-function (GOF) mutations in the Signal Transducer and Activator of Transcription 1 (STAT1) gene play a crucial role in predisposing individuals to chronic mucocutaneous candidiasis (CMC) caused by *C. albicans*, with a subsequent increased risk of oral cancers including OSCC. STAT1 GOF mutations result in hyperactivation of STAT1, intensifying cellular responses to interferons (IFN-α/β/γ) and Interleukin-27 (IL-27). This dysregulated signaling impairs differentiation of IL-17-producing T helper 17 (Th17) cells, which are essential for mucosal antifungal immunity. The consequent reduction in Th17 cells leads to defective clearance of *C. albicans* and chronic mucosal candidiasis [25, 27, 29, 30].

Patients with STAT1 GOF mutations often experience persistent and treatment-resistant oral and esophageal candidiasis from early childhood. The chronic exposure to *C. albicans* induces sustained inflammatory responses, favoring a microenvironment conducive to malignant transformation. Studies indicate that these patients exhibit a higher incidence of SCCs in the oral cavity and esophagus compared to the general population. This increased susceptibility is likely due to chronic inflammation, DNA damage, and epithelial barrier disruption engendered by persistent fungal infections [30, 31]. Furthermore, immune dysfunction associated with STAT1 GOF mutations facilitates overgrowth of other pathogens including viruses and bacteria, amplifying inflammatory stress and diminishing tumor surveillance in the mucosal tissues. Some reports suggest that the synergistic effect of chronic *Candida* infection and STAT1-mediated immune dysregulation elevates expression of pro-inflammatory mediators, adhesion molecules, and factors linked to cellular invasion and metastasis, thereby promoting oral carcinogenesis [29–31].

Moreover, Th17 immune responses, characterized by the secretion of IL-17 and IL-23 cytokines, are critical for antifungal defense against *C. albicans*. However, in the tumor microenvironment, elevated IL-17 promotes recruitment of tumor-associated macrophages (TAMs) that suppress the antitumor immune response, enhance cancer cell proliferation, angiogenesis, and tumor progression. This dual role of Th17 cytokines links fungal infection with the promotion of OSCC [20, 32–35].

## 4. Virulence Factors Related to *C. albicans* Carcinogenesis

**Figure 2:**
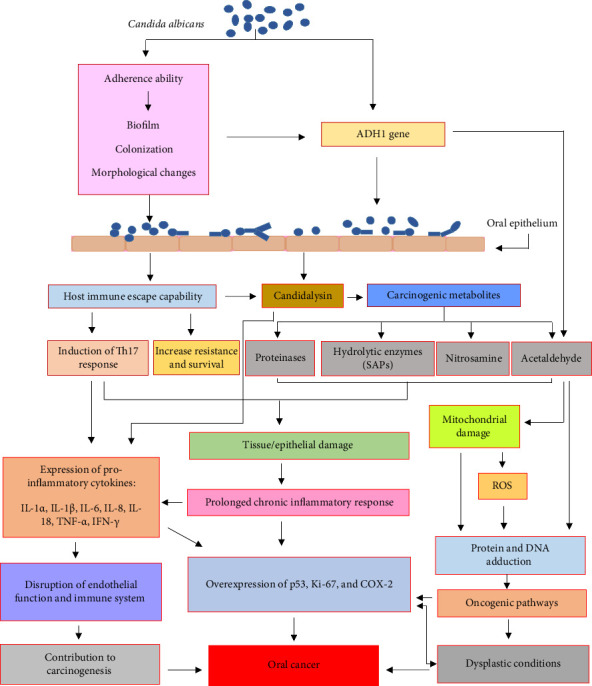
Pathways of *C. albicans* leading to oral cancer.

*Candida* spp. have been proven to cause a wide range of acute and chronic infections, especially in immunocompromised patients. Under certain conditions, their overgrowth can lead to pathogenicity and result in candidiasis [23, 26] (Figure 2).

### 4.1. Adhesion Ability

The adhesion ability of *C. albicans* is one of its most important pathogenic factors, facilitating colonization, biofilm formation, and evasion of the immune system. Following adhesion, yeast overgrowth is strongly associated with damage to host cells and oral epithelial dysplasia, which can contribute to the initiation of the OSCC process. [23]. The adhesion ability of *C. albicans* is attributed to its cell surface hydrophobicity (CSH). Surface glycosylation of *Candida* mannoproteins contributes to the hydrophobicity of the fungal cell membrane, thereby enhancing its ability to adhere to host cells [32]. Additionally, the presence of the agglutinin-like sequence (ALS) family, particularly ALS3, plays a significant role in this adhesion process [8, 37, 38]. ALS3 plays a crucial role in *C. albicans* adhesion to host cells and invasion by binding to specific receptors such as E-cadherin and N-cadherin. ALS3 mediates the endocytosis of the fungus by host cells, functioning not only as an adhesin but also as an invasin. This process facilitates immune evasion, tissue damage, apoptosis, necrosis, and disruption of the host's epithelial defenses, leading to structural changes that are precursors to cancer development. *C. albicans* also produces a peptide toxin called candidalysin, which induces calcium influx into host cells. This calcium influx triggers oxidative stress and mitochondrial dysfunction, resulting in adenosine triphosphate (ATP) depletion and cell necrosis. Furthermore, *C. albicans* influences key signaling pathways involved in cell proliferation and survival, such as the Mitogen-Activated Protein Kinase (MAPK) and Ras-Protein Kinase A (Ras-PKA) pathways, which can promote abnormal cell growth patterns characteristic of dysplasia. Chronic infection and sustained tissue damage lead to increased cell proliferation as a compensatory response. Over time, these hyperplastic changes may progress to dysplasia. Therefore, prolonged exposure to *C. albicans* may increase the risk of neoplastic transformation in epithelial tissues. This association is particularly evident in OSCC, where chronic candidiasis is linked to dysplastic lesions with potential progression to malignancy. Studies in this field emphasize the significant role of *C. albicans* in the development of oral epithelial dysplasia and its possible contribution to the progression of lesions to OSCC [26, 39].

### 4.2. Secreted Aspartyl Proteases (SAPs) and Candidalysin

SAPs are a family of enzymes produced by *C. albicans* that play a critical role in host tissue damage by degrading membrane proteins. These enzymes modify host cell surfaces by digesting membrane components and extracellular matrix proteins, thereby enhancing *C. albicans* adhesion and facilitating deeper tissue invasion. This proteolytic activity is especially important during the early stages of infection when adhesion to epithelial cells is essential for subsequent invasion [40, 41]. In addition to their direct tissue-destructive effects, SAPs potentiate the cytolytic activity of candidalysin. Candidalysin inserts into the lipid bilayer of host cell membranes, forming pores that cause leakage of cytoplasmic contents, cell lysis, and death. SAP-mediated degradation of host membrane components facilitates the insertion and hemolytic activity of candidalysin, amplifying epithelial damage [42, 43].

Candidalysin exerts multiple carcinogenesis-related effects, including inducing chronic inflammation, epithelial injury, proto-oncogene activation, and production of carcinogenic metabolites. It activates the NLRP3 inflammasome in macrophages, leading to secretion of pro-inflammatory cytokines such as IL-6 and IL-17, which promote a chronic inflammatory environment known to contribute to cancer progression [44, 45]. Candidalysin also directly induces cellular stress in epithelial cells, evidenced by increased double-strand breaks (DSBs) in DNA detected via γ-H2AX, a marker of DNA damage. It inhibits DNA repair by binding to cyclin-dependent kinase-activating kinase (CAK), impairs mitochondrial function, and triggers oxidative stress that elevates reactive oxygen species (ROS) production, all contributing to genomic instability [46]. Furthermore, candidalysin activates matrix metalloproteinases (MMPs), especially MMP-9, facilitating tumor invasion, angiogenesis, and metastasis by degrading the extracellular matrix and stimulating angiogenic factors such as vascular endothelial growth factor (VEGF) [47–49]. Moreover, *C. albicans* produces carcinogenic metabolites like acetaldehyde, which further contribute to DNA damage and mutation induction.

Besides tissue damage, SAPs aid immune evasion by degrading immune components such as complement proteins and other mediators, allowing *C. albicans* to evade host immune responses and persist within the host [40, 50] (Table 1).

### 4.3. Evasion of Host Immunity

*C. albicans* employs several mechanisms to evade the host immune system, including modifications in its cell wall structure, hyphal elongation, secretion of candidalysin, and modulation of immune responses such as the Th17 pathway [42, 48, 49]. These strategies help the fungus avoid immune recognition and elimination, resulting in persistent infections that create a chronic inflammatory microenvironment. This environment promotes immune suppression and tissue damage, facilitating the initiation and progression of oral carcinogenesis [8, 17, 51].

### 4.4. Structure of the Cell Wall

It has been shown that zymosan, a component derived from the cell wall of fungi such as *C. albicans*, significantly affects the cellular behavior of OSCC [8, 52]. Research indicates that zymosan stimulates the proliferation of OSCC cells through the Toll-like receptor 2 (TLR2)/MyD88/nuclear factor kappa B (NF-κB) signaling pathway. Initially, zymosan binds to TLR2 on OSCC cells, initiating a signaling cascade that activates MyD88, which in turn activates NF-κB. This activation leads to increased cell proliferation and enhanced inflammatory responses [53]. This activation subsequently results in enhanced expression of E-cadherin, which increases the adhesion of *C. albicans* to OSCC cells and induces the production of the pro-inflammatory cytokine IL-1β [53]. Zymosan also enhances IL-1β secretion in OSCC cells through the NLRP3/IL-1β pathway, an inflammatory response that may contribute to tumor progression and potentially affect treatment outcomes [8]. A study demonstrated that zymosan treatment significantly promotes the growth of various OSCC cell lines, including WSU-HN4, WSU-HN6, and CAL27. [53]. The interaction between *C. albicans* and OSCC cells, mediated by zymosan, reveals a complex relationship in which fungal components not only enhance cancer cell proliferation but also amplify inflammatory responses, potentially exacerbating cancer progression. This underscores the potential role of microbial components in influencing cancer biology and highlights the need for further research into therapeutic strategies targeting these interactions.

### 4.5. Hyphal Elongation

The elongation of hyphae in *C*. *albicans* is closely associated with the progression of OSCC. The morphological transition of *C. albicans* from yeast to hyphal form, a key virulence factor, enables the fungus to invade host tissues more effectively, resulting in increased colonization and persistence within the oral cavity. Hyphal forms of *C. albicans* can elicit significant inflammatory responses in host tissues, characterized by the release of pro-inflammatory cytokines, thereby creating a tumor-promoting microenvironment. Moreover, the presence of hyphal *C. albicans* has been shown to induce epithelial–mesenchymal transition (EMT) in OSCC cells, enhancing their migratory and invasive capabilities. Hyphal elongation also stimulates the production of MMPs in OSCC cells, which are critical for extracellular matrix remodeling and facilitate tumor invasion. These interactions are further linked to metabolic alterations that support tumor growth, including changes in the levels of key metabolites and the activation of protumor signaling pathways. Collectively, this multifaceted relationship between *C. albicans* and OSCC underscores the importance of understanding fungal infections in the context of cancer biology and highlights potential therapeutic strategies targeting both fungal infection and OSCC progression [49].

### 4.6. Production of Carcinogenic Metabolites

*C. albicans* can produce carcinogens such as acetaldehyde and nitrosamines that can cause cancer [54]. *C. albicans* has a greater potential than other *Candida* spp. to produce nitrosamine, which then converts nitrite to other substances to produce acetaldehyde [55, 56]. Nitrosamines disrupt the PI3K-Akt and Erk-MAP kinase signaling pathways, ultimately leading to mutagenesis in genes such as phosphatase and tensin homolog (PTEN), which are critical for regulating the PI3K-Akt pathway. When nitrosamines induce mutations or epigenetic changes that inactivate PTEN, this leads to sustained activation of Akt, promoting cell proliferation and survival while inhibiting apoptosis. Additionally, by disrupting the Erk-MAP kinase pathway, which is essential for mediating cellular responses to growth factors, nitrosamines can cause uncontrolled cell proliferation and differentiation, further contributing to carcinogenesis.

Furthermore, nitrosamines alter CYP450 expression and increase the accumulation of ROS. Elevated levels of ROS lead to increased DNA damage and mitochondrial dysfunction [56]. Moreover, acetaldehyde, by acting on enzymes involved in cytosine methylation and DNA repair such as DNA methyltransferases (DNMTs), Ten–Eleven Translocation (TET) family enzymes, and DNA deaminases, can cause proto-oncogene activation, cell cycle disorders, and tumor formation [13, 56]. Since the association of nitrosamines with OSCC in smokers has been proven, it can be concluded that nitrosamines produced by *C. albicans* also play a role in the development of oral cancers [10, 21, 55].

### 4.7. Role of *C. albicans* in Tumor Cell Adhesion

In healthy conditions of the human body, the pro-inflammatory response is crucial not only for the organization of the host's primary response to infection but also for the activation and recruitment of various immune cells [17]. However, in cancer patients, usually following chemotherapy, their immune system is suppressed, and the population of leukocytes decreases or sometimes even disappears [6, 25]. Therefore, circulating tumor cells stick to the endothelium instead of absorbing leukocytes and cause secondary tumors to become metastases. The results have shown that *C. albicans* can stimulate tumor metastasis by increase in the expression of adhesion molecules [17, 25]. Alcohol dehydrogenase (ADH), one of the most important proteins identified in *C. albicans* that increases tumor adhesion, is encoded by seven genes [57]. ADH1 is related to the production of acetaldehyde and causes the production of carcinogenic acetaldehyde [57]. Studies show that *C. albicans* strains isolated from oral cancer patients have a greater ability to produce acetaldehyde than healthy individuals, which can point to the possible role of *C. albicans* in increasing the incidence of oral cancer [13]. Acetaldehyde binds to proteins and DNA and reduces antioxidant activity [13, 56].

### 4.8. Inflammation and Carcinogenesis

Chronic inflammation driven by *C. albicans* is a key contributor to oral carcinogenesis. The persistent production of pro-inflammatory cytokines (IL-1β, IL-6, TNF-α) and MMPs (especially MMP-9) alters the extracellular matrix, promotes EMT, and supports tumor invasion and metastasis. Additionally, inflammatory mediators such as prostaglandin E2 (PGE2) facilitate tumor growth by suppressing antitumor immunity and inducing angiogenesis. These inflammation-driven molecular changes constitute a hallmark of *C. albicans*-associated oral cancer development [19, 38, 45, 51, 53, 57, 61].

### 4.9. Overexpression of p53, Ki-67, and COX-2

*C. albicans* can affect the overexpression of key biomarkers such as p53, Ki-67, and COX-2 through its interactions with host immune responses and inflammatory pathways in various ways. Following a strong inflammatory response in the host and the release of cytokines and growth factors, the expression of COX-2 is enhanced, which is associated with increased production of prostaglandins that stimulate cell proliferation and survival, contributing to dysplasia and tumor progression. Additionally, the overexpression of COX-2 in *C. albicans* can also exacerbate inflammation and create a chronic inflammatory state by modulating immune responses, particularly through reducing the activity of immune cells such as macrophages and lymphocytes. This is accompanied by an increase in the expression of adhesion molecules and invasive factors, which can allow *C. albicans* to evade the immune system, creating a favorable environment for its growth, proliferation, and spread [26, 62]. Moreover, the presence of *C. albicans* can increase the Ki-67 labeling index, a marker for cellular proliferation. This increase indicates that *C. albicans* may stimulate rapid cell division in epithelial tissues, often serving as a precursor to dysplastic changes and malignancy. On the other hand, the overexpression of Ki-67, a marker associated with cellular proliferation, significantly impacts the growth patterns of *C. albicans*. Elevated levels of Ki-67 in host cells can enhance epithelial cell turnover, thereby creating a conducive environment for fungal colonization. While initially helping to control the growth of *C. albicans*, this overexpression may lead to chronic inflammation and tissue damage, ultimately facilitating fungal persistence. In cancer patients, high levels of Ki-67 may indicate dysplastic changes in epithelial tissues that alter the tumor microenvironment and increase susceptibility to infections and treatment-related complications. Furthermore, increased Ki-67 is associated with heightened cytokine production, exacerbating inflammatory responses against *C. albicans* and promoting its growth through inflammation and cycles of cellular proliferation. Excessive Ki-67 may encourage EMT, facilitating the invasion of *C. albicans* into altered tissues [26]. The role of p53 is crucial in tumor suppression. However, studies have shown that persistent infections such as those caused by *C. albicans* can lead to mutations or functional changes in p53, potentially resulting in overexpression or loss of function. This dysregulation can disrupt normal apoptotic responses and allow uncontrolled cell growth. Thus, *C. albicans*, along with the overexpression of p53, Ki-67, and COX-2, can enter a vicious cycle that amplifies their effects on each other [26, 61, 63, 64].

## 5. Conclusion

The role of *C. albicans* in oral carcinogenesis has increasingly garnered attention, with evidence suggesting that this opportunistic pathogen can contribute to the development of OSCC through various mechanisms. This yeast is capable of producing carcinogenic metabolites and inducing chronic inflammation, leading to cellular damage and mutations. While *C. albicans* is not the sole cause of oral cancer, its presence alongside traditional risk factors such as tobacco and alcohol use may synergistically elevate cancer risk. The interaction between *C. albicans* and other oral microorganisms may further enhance its pathogenic potential, underscoring the importance of the oral microbiome in cancer risk. Research has shown that this yeast can significantly damage mucosal tissues through the production of enzymes and toxic metabolites, triggering inflammatory responses that promote the proliferation and resistance of precancerous and cancerous cells to apoptosis and stress. Therefore, a deeper understanding of these complex interactions is essential for developing effective prevention and treatment strategies. Future research should focus on elucidating the specific molecular mechanisms by which *C. albicans* contributes to oral carcinogenesis. Such investigations could lead to the identification of new therapeutic approaches aimed at mitigating the impact of *C. albicans* on oral health. Additionally, a more thorough examination of the relationship between *C. albicans* and oral cancer could provide new insights for prevention, diagnosis, and treatment strategies for oral cancers. Ultimately, considering the roles of environmental factors, host immunity, and underlying diseases is crucial for a comprehensive understanding of this phenomenon.

## Figures and Tables

**Table 1:** Roles of SAPs and candidalysin in carcinogenesis.

Factor	Role in carcinogenesis	Key mechanisms and effects	Reference
SAPs	Host tissue degradation and enhanced fungal invasion	Proteolytic degradation of host membranes and extracellular matrix, immune evasion	[40, 41, 50]
Candidalysin	Induction of chronic inflammation and genomic instability	Membrane pore formation, inflammasome activation, DNA damage, activation of MMP-9, and production of carcinogenic metabolites	[33, 42, 45–47]

*Note:* MMP-9: Matrix Metalloproteinase-9.

## Data Availability

No new data were generated or analyzed in this study. All data discussed are available in the cited literature.
